# Profiling of Sexually Dimorphic Genes in Neural Cells to Identify *Eif2s3y*, Whose Overexpression Causes Autism-Like Behaviors in Male Mice

**DOI:** 10.3389/fcell.2021.669798

**Published:** 2021-07-06

**Authors:** Muxian Zhang, Yunqiang Zhou, Yiru Jiang, Zhancheng Lu, Xiaoxia Xiao, Jinhuan Ning, Hao Sun, Xian Zhang, Hong Luo, Dan Can, Jinsheng Lu, Huaxi Xu, Yun-wu Zhang

**Affiliations:** ^1^Fujian Provincial Key Laboratory of Neurodegenerative Disease and Aging Research, Institute for Neuroscience, School of Medicine, Xiamen University, Xiamen, China; ^2^Emergency Department, Xiang’an Hospital, Xiamen University, Xiamen, China; ^3^Institute of Chemistry, University of Vienna, Vienna, Austria; ^4^Xiamen Key Laboratory of Brain Center, The First Affiliated Hospital of Xiamen University, Xiamen, China

**Keywords:** autism, *Eif2s3y*, immune system, sex differences, sexually dimorphic genes, Y chromosome

## Abstract

Many neurological disorders exhibit sex differences and sex-specific therapeutic responses. Unfortunately, significant amounts of studies investigating molecular and cellular mechanisms underlying these neurological disorders use primary cell cultures with undetermined sexes; and this may be a source for contradictory results among different studies and impair the validity of study conclusion. Herein, we comprehensively compared sexual dimorphism of gene expression in primary neurons, astrocytes, and microglia derived from neonatal mouse brains. We found that overall sexually dimorphic gene numbers were relatively low in these primary cells, with microglia possessing the most (264 genes), neurons possessing the medium (69 genes), and astrocytes possessing the least (30 genes). KEGG analysis indicated that sexually dimorphic genes in these three cell types were strongly enriched for the immune system and immune-related diseases. Furthermore, we identified that sexually dimorphic genes shared by these primary cells dominantly located on the Y chromosome, including *Ddx3y*, *Eif2s3y*, *Kdm5d*, and *Uty*. Finally, we demonstrated that overexpression of *Eif2s3y* increased synaptic transmission specifically in male neurons and caused autism-like behaviors specifically in male mice. Together, our results demonstrate that the sex of primary cells should be considered when these cells are used for studying the molecular mechanism underlying neurological disorders with sex-biased susceptibility, especially those related to immune dysfunction. Moreover, our findings indicate that dysregulation of sexually dimorphic genes on the Y chromosome may also result in autism and possibly other neurological disorders, providing new insights into the genetic driver of sex differences in neurological disorders.

## Introduction

Numerous neurological disorders display a prominent sexual dimorphism, such as Alzheimer’s disease (AD), Parkinson’s disease (PD), schizophrenia, autism, and multiple sclerosis (Aleman et al., 2003; Orton et al., 2006; Haaxma et al., 2007; Werling and Geschwind, 2013; Mielke et al., 2014; Maldonado Weng et al., 2019). For example, it is well-known that men are more vulnerable to autism and autism spectrum disorders (ASDs) than women, with a prevalence ratio of 4∼5:1 (Fombonne, 2009; Christensen et al., 2016). Elucidating the mechanistic underpinnings are important not only for understanding the sex-biased susceptibility of these devastating diseases but also for sex-specific treatment purposes.

Sex differences in disease prevalence and manifestation are rooted in the genetic sex differences, which are genetic females XX or genetic males XY in most mammals. The primary testicular/ovarian gonadal phenotype is determined by the sex-determining region of the Y chromosome (*SRY*) gene during embryonic development. Sex hormones (e.g., androgens and estrogens) and their receptors (e.g., androgen and estrogen receptors) have traditionally been considered the major cause of sex differences in the brain. However, emerging evidence has indicated that sex chromosome effects also contribute to the brain sex differences throughout the lifespan, independent of sex hormones (Arnold, 2017). Sex chromosome aneuploidies (SCAs) are characterized by an abnormal number of X or Y chromosome (Ngun et al., 2011; Hong and Reiss, 2014). Turner Syndrome (45,X), Klinefelter Syndrome (47,XXY), and XYY Syndrome (47,XYY) are the most common SCAs (Heard and Turner, 2011). Clinical characteristics of SCAs often include intellectual disability, motor impairments, and an increased risk of neurological and psychiatric disorders such as ASDs, demonstrating that genes on sex chromosomes are important for maintaining normal brain functions (Rovet et al., 1995; Visootsak and Graham, 2009; Leggett et al., 2010; Tartaglia et al., 2010; Bishop et al., 2011; Hong et al., 2011; Hong and Reiss, 2012, 2014; Printzlau et al., 2017; Green et al., 2019). Indeed, mutations in multiple X-chromosomal genes have been found to cause X-linked intellectual disability and ASDs (Schaafsma and Pfaff, 2014; Fung and Reiss, 2016). Patients carrying more than one Y chromosome also exhibit ASD symptoms (Demily et al., 2017; Tartaglia et al., 2017; Matsuzaki et al., 2019), suggesting that abnormality of Y-chromosomal genes may also result in ASD. However, the underlying mechanism remains largely elusive.

Much of our understanding of cellular and molecular mechanisms underlying neurological disorders are based on research using primary neural cells derived from animals at embryonic or neonatal developmental stages. Unfortunately, the sex of donors of these primary cells is not reported in most published studies. In many cases, the cells used in experiments are a mixture of both sexes. This could potentially jeopardize the conclusion of these studies, especially when genetic sex differences play a significant role in such disorders.

Therefore, a major aim of the present study is to determine whether the transcriptome of primary neurons, astrocytes, and microglia isolated from neonatal mice exhibit any significant sex difference. Herein, we found that overall sexually dimorphic gene numbers were low in these primary cells, and sexually dimorphic genes were enriched for the immune system and immune-related diseases. In addition, sexually dimorphic genes shared by all the three cell types dominantly located on sex chromosomes; and one of them is *Eif2s3y*.

*Eif2s3y*, a gene located on the non-recombining region of the mouse Y chromosome, is essential for normal spermatogonia proliferation and differentiation (Mazeyrat et al., 2001; Yamauchi et al., 2014; Matsubara et al., 2015). *Eif2s3y* is ubiquitously expressed in various tissues (Wolstenholme et al., 2013). In the brain, *Eif2s3y* is preferentially expressed in specific regions including the hippocampus, thalamus, striatum, and olfactory areas (Xu et al., 2006). Prior to gonadal differentiation (∼E12–E14), *Eif2s3y* is highly expressed in male mouse brain; and this sexual dimorphism expression can also be observed in the stage of gonadal hormone secretion phase (E18.5) and endures to adulthood (Wolstenholme et al., 2013). However, the function of *Eif2s3y* in the brain has not yet been reported. In the current study, we demonstrated that overexpression of *Eif2s3y* altered synaptic transmission specifically in male neurons and led to autism-like behaviors specifically in male mice.

## Materials and Methods

### Animals

All wild-type C57BL/6J mice were housed under Specific-Pathogen-Free (SPF) conditions in the core animal facility at the Xiamen University. Mice were maintained on an 8:00–20:00 light/dark cycle with *ad libitum* access to phytoestrogen-free chow and water. Pregnant females were housed separately. All animal procedures were approved by the Animal Ethics Committee of Xiamen University and were conducted in accordance with the National Institutes of Health Guidelines for the Care and Use of Laboratory Animals.

### Antibodies

Anti-Myc (2276S), anti-β-actin (8457S), anti-GluR1 (13185S), anti-NR1 (5704S), anti-PSD-95 (3450S), and anti-Map2 (4542S) antibodies are from Cell Signaling Technology. Anti-GFAP (16825-1-AP), anti-GluR2 (11994-1-AP), anti-NR2A (19953-1-AP), and anti-NR2B (19954-1-AP) antibodies are from Proteintech. Anti-Iba1 (019-19741), anti-GFP (M20004L), anti-NeuN (ab177487), anti-GluR3 (MAB5416), and anti-synaptophysin (S5768) antibodies are from Wako, Abmart, Abcam, Millipore, and Sigma, respectively. HRP-conjugated goat anti-mouse IgG (H + L) secondary antibody (31430), HRP-conjugated goat anti-rabbit IgG (H + L) secondary antibody (31460), Alexa fluor 488-conjugated donkey anti-mouse IgG (H + L) secondary antibody (A-21202), Alexa fluor 488-conjugated goat anti-rabbit IgG (H + L) secondary antibody (A-11008), and Alexa fluor 594-conjugated goat anti-rabbit IgG (H + L) secondary antibody (A-11012) are from Thermo Fisher Scientific.

### Primary Neuron, Astrocyte, and Microglia Cultures

Wild-type C57BL/6J mice at postnatal day 0 (P0) were euthanized within 12 h after birth. The brain was dissected and placed in ice-cold Hanks’ Balanced Salt Solution (HBSS; Gibco). After stripping off meninges, half of the cerebrum (including cortex and hippocampus) was used for preparing neuron cultures, and the other half of the cerebrum plus the entire cerebellum was used for preparing astrocyte and microglia cultures.

For neuron cultures, half of the cerebrum was dissociated in HBSS with a pipette and then incubated with an enzymatic digestion solution containing 0.25% trypsin-EDTA (Gibco) and DNase I (0.2 kU/mL, Worthington) at 37° for 15 min. After centrifuging at 1000 rpm for 5 min, cell pellets were re-suspended in neurobasal medium (Gibco) supplemented with 2% B-27 (Gibco), 2 mM L-glutamine (Gibco), and 100 U/mL penicillin and 100 μg/mL streptomycin (Gibco). Cell density was counted using TC20 Automated Cell Counter (Bio-Rad). Primary neurons were seeded in an appropriate poly-L-lysine coated dish, cultured for 12 days *in vitro* (DIV) in a 37° incubator with 5% CO_2_, and used for RNA sequencing.

For astrocyte and microglia cultures, brain samples were triturated with a 5 mL pipette and then seeded in a poly-L-lysine coated T75 flask with microglia culture media containing DMEM (Gibco), 10% heat-inactivated fetal bovine serum (FBS; Gibco), and 100 U/mL penicillin and 100 μg/mL streptomycin. After incubating in a 37° incubator with 5% CO_2_ for 24 h, culture media were replaced with new microglia culture media and supplemented with 25 ng/mL mGM-CSF (R&D Systems). On DIV11, the flask was shaken on a rotating platform at 200 RPM for 20 min. Microglia floated in the media were collected by centrifugation and then re-suspended and cultured separately in microglia culture media for another day before subjected to RNA sequencing. The flask was refilled with culture media and incubated for another 3 days. After shaking off and removing newly proliferated microglia, the flask was refilled with fresh astrocyte culture medium (DMEM + 10% regular FBS + 100 U/mL penicillin + 100 μg/mL streptomycin) to prevent microglia proliferation. 3 days later, unattached cells were shaken off and discarded. Astrocytes were detached from the flask bottom by 0.25% trypsin digestion and then re-suspended and cultured in astrocyte culture media for another day before subjected to RNA sequencing.

### Sex Identification

Mouse sex was identified by PCR. Briefly, genomic DNA was isolated from mouse tails using NaOH extraction and subjected to nested PCR using the primer pair: Sex-F (5′-ATGTGTTCCCGTGGTGAGAG-3′) and Sex-R (5′-GGTCGTCCAGTTTTGTTGAG-3′).

### RNA Sequencing and Analysis

Cultured primary neurons, astrocytes, and microglia were soaked in the TRIzol reagent (Life Technologies) and transferred to Beijing Genomics Institute. RNA extraction, cDNA library preparation, and RNA sequencing using the BGISEQ-500 platform were conducted by Beijing Genomics Institute with standard protocols. Generated raw sequencing reads were filtered by using SOAPnuke^1^ to remove reads with adaptors, reads with more than 5% of unknown bases, and low-quality reads. Clean reads were aligned to *Mus musculus* reference genes (reference genome version: GCF_000001635.25_GRCm38.p5) using Bowtie2 (version 2.2.5) (Langmead and Salzberg, 2012). Gene expression levels were calculated and normalized to fragments per kilobase of transcript per million mapped reads (FPKM) using the RSEM software (version 1.2.12) (Li and Dewey, 2011) with default parameters. Principal component analysis (PCA), which converts the data into a few independent metagenes by dimensionality reduction analysis, was carried out using the *princomp* function in the R software package. Pearson’s correlation analysis between samples was carried out using the *cor* function in the R software package. Differentially expressed genes (DEGs) between males and females were determined using DEGseq (Wang et al., 2010), with a fold change ≥ 2 and false discovery rate (FDR) adjusted *P*-value ≤ 0.001 considered to be differentially expressed. DEG lists were annotated with the Gene Ontology (GO) terms and Kyoto Encyclopedia of Genes and Genomes (KEGG) pathways.

### Quantitative Real-Time PCR (qRT-PCR)

Total RNAs were extracted using the TRIzol reagent. One microgram RNA was used for cDNA synthesis using Rever Tra Ace qPCR RT Master Mix (TOYOBO). qRT-PCR was performed on the LightCycler 480 II instrument (Roche) with FastStart Universal SYBR Green Master (ROX) (Roche). The primers used are shown in Supplementary Table 1. Expression levels of target genes were normalized to those of the control gene β-actin for comparison.

### Adeno-Associated Virus (AAV) Infection

AAV2/9 (serotype 2/9) viruses carrying mouse Eif2s3y-Myc-P2A-EGFP or control EGFP were packaged by OBIO Technology (Shanghai, China). Cultured primary neurons were infected with AAV on DIV 5, and electrophysiology was carried out on DIV12-13.

For *in vivo* injection, P0 C57BL/6J wild-type mice were subjected to hypothermic anesthesia. Then one microliter virus (1.55 × 10^13^ V.G./mL) was slowly injected into each lateral ventricle (2 mm distance from ventral to skin and 2/5 from lambda suture to the eye). Injected mice were laid on a warming pad until the body temperature was fully recovered.

### Immunostaining

Primary neurons, astrocytes, and microglia were fixed in 4% paraformaldehyde and permeabilized in 0.2% Triton X-100 before blocking in 5% BSA for 1 h at room temperature. Cells were incubated with indicated primary antibodies overnight at 4°C, and then incubated with appropriate secondary antibodies conjugated with fluorescence and DAPI for 1 h at room temperature. Images were captured using the A1R (Nikon) confocal microscope.

Mice injected with AAV were anesthetized and perfused with PBS from the heart. The brain was dissected quickly, post-fixed in 4% paraformaldehyde at 4°C for 24 h, and cryoprotected in 30% sucrose. After sectioning using a freezing microtome (Leica), brain sections (15 μm thickness) were blocked in 0.2% Triton X-100 and 5% BSA for 1 h at room temperature and then incubated with indicated primary antibodies overnight at 4°C, followed by incubation with appropriate secondary antibodies conjugated with fluorescence for 2 h at room temperature. Images were captured with the Aperio Versa 200 (Leica), the FV1000MPE-B (Olympus), or the LSM 880 (Carl Zeiss) microscope.

### Western Blot

Mouse brain samples were lysed in the TNEN lysis buffer (50 mM Tris-HCl, pH 8.0, 150 mM NaCl, 2 mM EDTA, and 1% NP-40, supplemented with protease inhibitors and phosphatase inhibitors). Protein lysates (30 μg) were subjected to SDS-polyacrylamide gel electrophoresis. After being transferred to PVDF membranes, protein samples were incubated with indicated primary antibodies overnight at 4°C, with appropriate HRP-conjugated secondary antibodies for 1 h at room temperature, and then with enhanced chemiluminescent reagent (Millipore) for protein band development. Protein band intensities were quantified by the Image J software for comparison.

### Preparation of Synaptosomal and Postsynaptic Density Fractions

The hippocampal tissues of male mice injected with AAV were dissected and homogenized in ice-cold sucrose buffer (0.32 M sucrose, and 25 mM HEPES, pH 7.4, supplemented with protease inhibitors and phosphatase inhibitors). The homogenate was centrifuged at 300 g for 5 min to separate the supernatant (S1) and the pellet (P1, nuclei and large debris fraction). The P1 fraction was discarded and the S1 fraction was centrifuged for 12 min at 10,000 g to separate the supernatant (S2, light membrane and cytosolic fraction) and the pellet (P2, crude synaptosomal fraction). The P2 fraction was washed twice by ice-cold sucrose buffer and re-suspended in ice-cold HBS buffer (150 mM NaCl, and 25 mM HEPES, pH 7.4, supplemented with protease inhibitors and phosphatase inhibitors) to get the synaptosomal (Syn) fraction. Partial Syn fraction was supplemented with ice-cold 1% Triton, incubated at 4°C for 30 min, and centrifuging at 20,000 *g* for 30 min. The resulting pellet was re-dissolved in 3% SDS HBS buffer to get the postsynaptic density (PSD) fraction.

### Whole-Cell Patch-Clamp Electrophysiology

Primary neurons infected with AAV containing Eif2s3y-Myc-P2A-EGFP or control EGFP were bathed in an artificial cerebrospinal fluid (aCSF) containing 126 mM NaCl, 2.5 mM KCl, 2.4 mM MgCl_2_⋅6H_2_O, 1.2 mM CaCl_2_, 1.2 mM NaH_2_PO_4_, 11 mM NaHCO_3_, and 18 mM glucose, pH 7.4, 290–300 mOsm, bubbled with 95% O_2_/5% CO_2_ (v/v). Infected neurons were selected with a microscope equipped with a GFP fluorescent filter set (Olympus), and visualized with differential interference contrast (DIC) at room temperature. Recording pipettes (5∼8 MΩ tip resistance) were filled with a solution containing 140 mM CsCH_3_SO_3_, 10 mM HEPES, 1 mM EGTA, 2 mM MgCl_2_⋅6H_2_O, 5 mM TEA-CL, 2.5 mM Mg-ATP, and 0.3 mM Na-GTP, pH 7.3, 280 mOsm. Spontaneous excitatory postsynaptic currents (sEPSCs) and spontaneous inhibitory postsynaptic currents (sIPSCs) were measured in the voltage-clamp mode at holding potential of -70 mV and 0 mV, respectively. Synaptic currents were amplified and filtered at 2 kHz (Multiclamp 700B, Molecular Devices) and digitized at 10 kHz (pClamp10.7/Axon Digidata 1550B, Molecular Devices). sEPSCs and sIPSCs were recorded for 5 min and analyzed with MiniAnalysis (Synaptosoft).

### Behavioral Analysis

All behavioral tests, except nesting, were performed between 9: 00 A.M. and 4: 00 P.M. in a blinded manner. Mice were acclimated to handling for 3 days before tests and to the test room for 1 h prior to tests. All behavioral tests, except nesting and self-grooming, were recorded and analyzed by a Smart 3.0 video tracking system (Panlab, Harvard Apparatus).

### Open-Field Test

Mice were placed in an open field box (40 cm (L) × 40 cm (W) × 40 cm (H)) and allowed to explore for 10 min freely. Their total travel distance and time spent in the center arena were analyzed.

### Three-Chamber Social Interaction Test

The three-chamber social interaction test was performed as described previously (Zheng et al., 2017). Briefly, mice were tested in three steps: (1) their time spent in each of the three empty chambers (left, center, and right) during a 10 min habituation; (2) their time spent interacting with an empty wire cage (E) in the right chamber versus a mouse (S1) in one wire cage in the left chamber during a 10-min sociability test; and (3) their time spent interacting with the familiar mouse (S1) versus an unfamiliar mouse (S2) put into the wire cage in the right chamber during a 10-min social novelty test.

### Nesting

Mice were individually placed in a home cage containing 2 cm bedding and cotton tissues (3 g) overnight. Nest quality was then scored on a 1-5 scale as described previously (Kim et al., 2017): 1 point, cotton tissue rarely touched; 2 points, cotton tissue partially ripped; 3 points, cotton tissue mostly torn; 4 points, cotton tissue shredded into flat nest shape (walls lower than the mouse); and 5 points, perfect nest shape (walls higher than the mouse). Nesting scores were manually analyzed in a blinded manner.

### Self-Grooming

Mice were individually placed in a home cage with no bedding. Following a 5 min habituation, mouse activity was videotaped for another 10 min. The number of bouts and total grooming duration were manually analyzed in a blinded manner.

### Elevated Plus-Maze Test

Mice were placed in the center area of an elevated plus-maze with two 30 cm (L) × 6 cm (W) open arms and two 30 cm (L) × 6 cm (W) × 15 cm (H) closed arms. The movement of mice was recorded for 5 min. The time spent in open arms and the number of open arm entries were analyzed.

### Morris Water Maze Test

The Morris Water Maze test was performed as described previously (Zheng et al., 2017). In brief, mice were trained for 5 consecutive days in a circular tank (90 cm (D) × 35 cm (H)) filled with tap water (made opaque by TiO2) at a temperature of 22°C. Contrasted shapes were posted on the walls to serve as reference cues. A hidden platform was placed in the southeast quadrant 1 cm below the water surface. Mice were released into the pool at one of four points (northwest, northeast, southwest, and southeast) in a random order to let them find and climb onto the hidden platform within 60 s. If a mouse failed to reach the hidden platform within the timeline, it was gently guided to the platform and allowed to stay on the platform for 10 s. Two trials per day were performed with at least 1 h inter-trial interval. On the 6th day, the hidden platform was removed from the pool, and mice were released into the pool for a 60 seconds’ probe test. Mouse escape latency during the training phase and quadrant entry time during the probe test phase were analyzed.

### Y-Maze Test

Mice were placed in the center area of a Y-shape maze with three symmetrical arms at 120° angles (30 cm (L) × 6 cm (W) × 15 cm (H)). Each mouse was allowed to explore the maze freely for 5 min. An alternation is defined as consecutive entries into three different arms. The alternations and the total arm entries were recorded. The percentage of alternation triplet was calculated using the following formula as previously described (Miedel et al., 2017; Lachance et al., 2019):

$$\mathrm{Alternation}\mathrm{triplet}\left(\%\right)=\left(\frac{\mathrm{Alternation}\,\mathrm{numbers}}{\mathrm{Total}\,\mathrm{arm}\,\mathrm{entries}-2}\right)\times 100$$

### Tail Suspension Test

Mice were suspended by the tail in an acrylic bar (15 cm (L) × 30 cm (H)) for 7 min. Total immobility duration during the last 6 min period was analyzed.

### Statistical Analysis

All statistical analysis was performed using Prism 8.3 (GraphPad Software, La Jolla, CA, USA). Statistical differences between two groups were assessed by unpaired t-test for normally distributed data or non-parametric Mann Whitney test for non-normally distributed data. Statistical differences between multiple groups were assessed by one-way ANOVA with *post hoc* Dunnett’s multiple comparisons test for non-normally distributed data, or two-way ANOVA with *post hoc* Sidak’s multiple comparisons test for normally distributed data. Data are plotted as mean ± standard error of mean (SEM). *p* < 0.05 was considered to be statistically significant.

## Results

### Identification of Sexually Dimorphic Genes in Neurons, Astrocytes, and Microglia of Neonatal Mouse Brains

**FIGURE 1:**
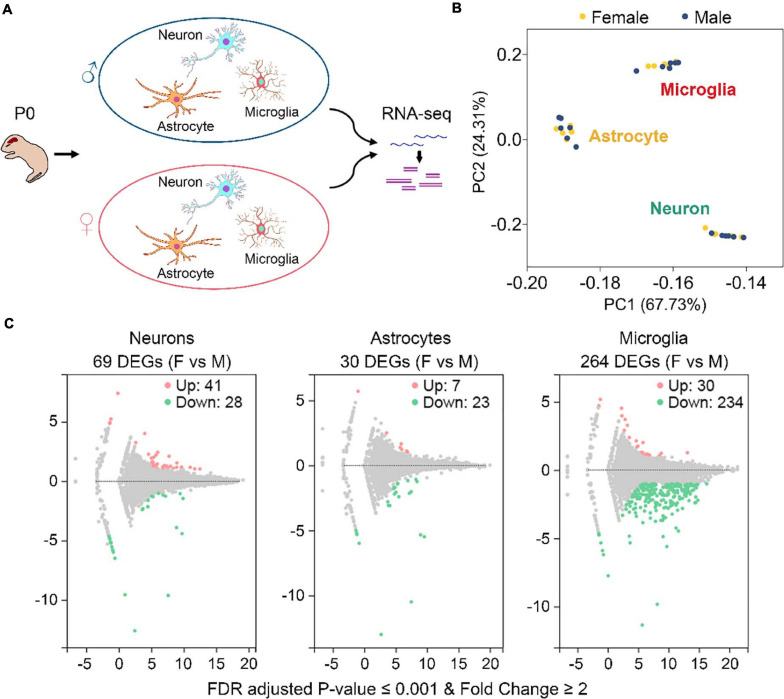
Identification of sexually dimorphic genes in neurons, astrocytes, and microglia. **(A)** Sample preparation strategy. Primary neurons, astrocytes, and microglia were isolated from the same postnatal day 0 (P0) male and female mice and cultured *in vitro*. RNAs were extracted from individual cultures and subjected to RNA sequencing. For each cell type, *n* = 6 males from two litters and *n* = 6 females from the same two litters. **(B)** PCA analysis of gene expression depicts three discrete subsets corresponding to neuron, astrocyte, and microglia samples, respectively. Yellow and blue dots represent female and male samples, respectively. **(C)** Volcano plots of gene expression change between female (F) and male (M) in neurons, astrocytes, and microglia. Pink and green dots represent genes that are expressed significantly higher and lower in females vs. males, respectively. Benjamini-Hochberg method, Storey and Tibshirani’s approach, false discovery rate (FDR) adjusted *P*-value ≤ 0.001, fold change ≥ 2. A total of 69, 30, and 264 differentially expressed genes (DEGs) were identified in neurons, astrocytes, and microglia, respectively.

To study gene expression profiling differences between sexes in neurons, astrocytes, and microglia, we collected 6 postnatal day 0 (P0) males and 6 P0 females from 2 C57BL/6J mouse litters (3 males and 3 females from each litter) (Figure 1A and Supplementary Figure 1A). These mice were euthanized within 12 h after birth. Primary neurons, astrocytes, and microglia were isolated from the same individual mouse brain and cultured *in vitro* (Figure 1A). Purities of each harvested cultures were determined to be over 90% (Supplementary Figure 1B). A total of 36 cell cultures were used for RNA sequencing, including 12 (6 males and 6 females) neuronal, 12 (6 males and 6 females) astrocytic, and 12 (6 males and 6 females) microglial cultures.

RNA sequencing generated about 21.94 million raw reads for each sample (Supplementary Table 2). After data filtering using the SOAPnuke software, an average of 21.33 million clean reads for each sample was acquired. Mapping the clean reads onto the mouse reference genes using the Bowtie2 software (Langmead and Salzberg, 2012) revealed an average mapping ratio of 86.48% (Supplementary Table 2). A total of 20,073 genes were identified from the 36 samples.

Gene expression levels, described as fragments per kilobase of transcript per million mapped reads (FPKM), were determined using the RSEM software (Li and Dewey, 2011). As expected, principal component analysis (PCA) revealed three discrete expression clusters consistent with the three different cell types (Figure 1B). Pearson’s correlation analysis also showed high correlations for samples within the same cell type (all *r* > 0.91) (Supplementary Figure 1C). However, neither PCA nor Pearson’s analysis distinguished male and female samples within each cell type, suggesting that gene expression differences between males and females are minimal compared to those between different cell types.

**FIGURE 2:**
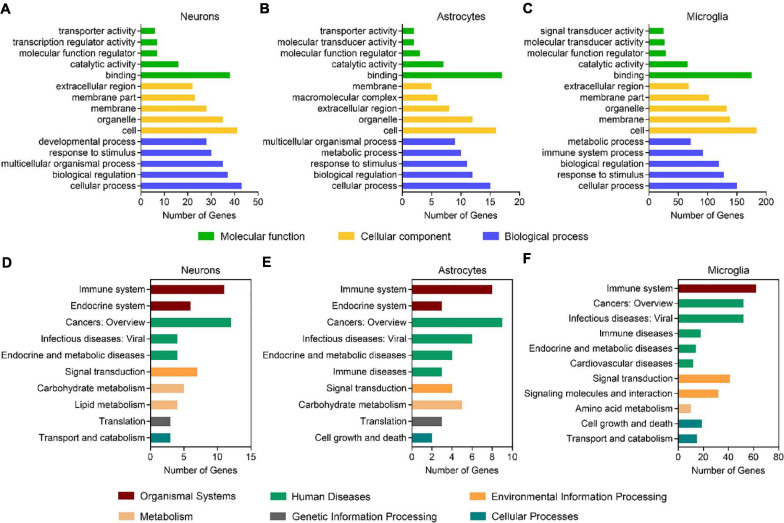
Sexually dimorphic genes exhibit similar annotation patterns in neurons, astrocytes, and microglia. **(A–C)** GO analysis of DEGs (male versus female) in neurons **(A)**, astrocytes **(B),** and microglia **(C)**. Green bars represent the molecular function, yellow bars represent the cellular component, and blue bars represent the biological process. **(D–F)** KEGG analysis of DEGs (male versus female) in neurons **(D)**, astrocytes **(E),** and microglia **(F)**. Red bars represent organismal systems, green bars represent human diseases, orange bars represent environmental information processing, flesh color bars represent metabolism, gray bars represent genetic information processing, and blue bars represent cellular processes.

By using the DEGseq software (Wang et al., 2010), we identified 69, 30, and 264 differentially expressed genes (DEGs, FDR adjusted P-value ≤ 0.001 and fold change ≥ 2) between males and females in neurons, astrocytes, and microglia, respectively (Figure 1C and Supplementary Table 3). Among them, 41 DEGs were upregulated and 28 DEGS were downregulated in female neurons versus male neurons, 7 DEGs were upregulated and 23 DEGS were downregulated in female astrocytes versus male astrocytes, and 30 DEGs were upregulated and 234 DEGS were downregulated in female microglia versus male microglia. We carried out Gene Ontology (GO) and KEGG pathway analysis and found that identified sexually dimorphic genes showed similar annotation patterns in neurons, astrocytes, and microglia. In GO analysis, sexually dimorphic genes in all the three cell types were strongly enriched for “binding” in the “molecular function” category, enriched for “cell” in the “cellular component” category, and enriched for “cellular process” in the “biological process” category (Figures 2A–C). In KEGG pathway analysis, sexually dimorphic genes in all the three cell types were strongly enriched for “immune system” in the “organismal system” category, enriched for “cancers: overview” and “infectious diseases: viral” in the “human diseases” category, and enriched for “signal transduction” in the “environmental information processing” category (Figures 2D–F).

### Sexually Dimorphic Genes Shared by Different Neural Cell Types Locate Dominantly on Sex Chromosomes

**FIGURE 3:**
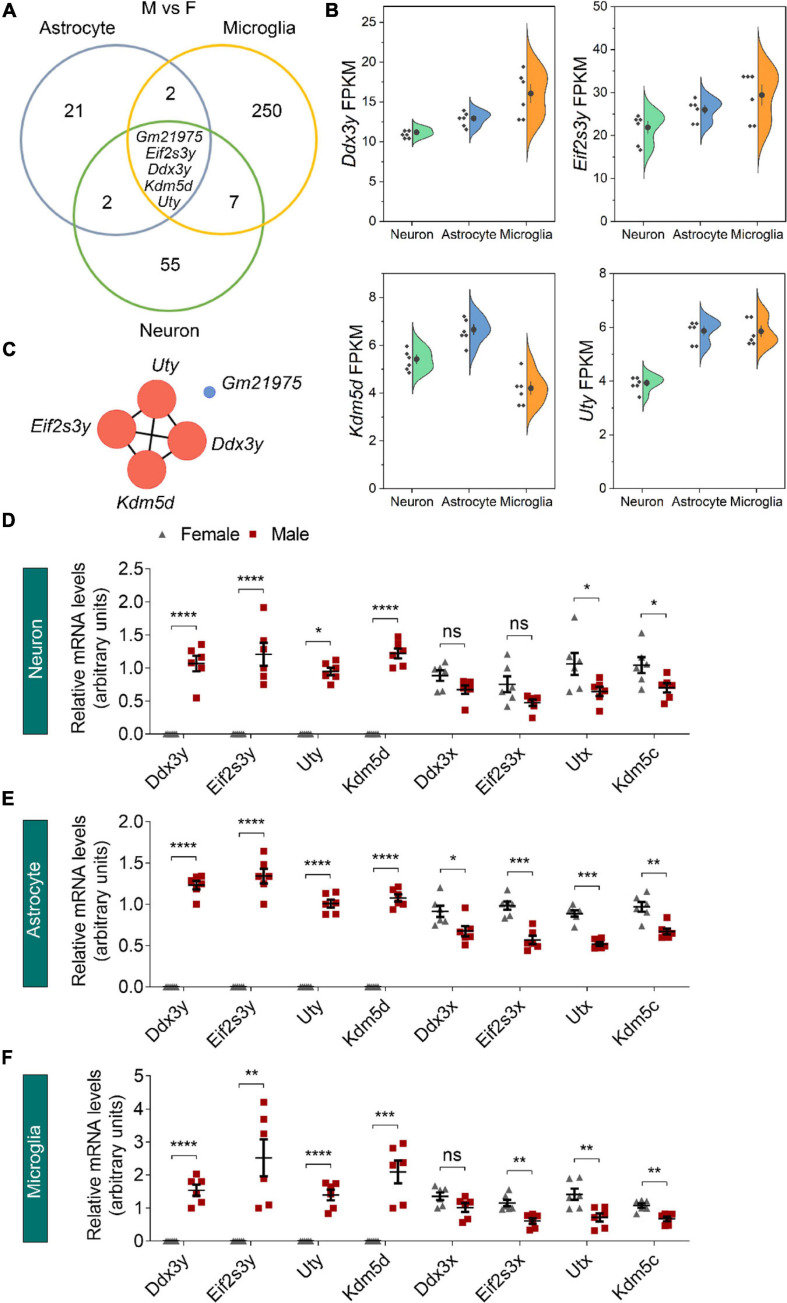
Identification of sexually dimorphic genes shared by different neural cell types. **(A)** Venn diagram analysis revealed five DEGs (male vs. female, M vs. F) shared by neurons, astrocytes, and microglia. **(B)** RNA sequencing identified *Ddx3y*, *Eif2s3y*, *Kdm5d*, and *Uty* expression levels (represented using FPKM) in male neurons, astrocytes, and microglia. *n* = 6 per group. **(C)** Protein-protein interaction analysis depicted a robust interaction within the four Y-linked genes: *Ddx3y*, *Eif2s3y*, *Kdm5d*, and *Uty.* **(D–F)** Relative mRNA levels of the four Y-linked genes and respective homologous genes on the X chromosome in neurons **(D)**, astrocytes **(E),** and microglia **(F)** were determined by qRT-PCR and compared between male and female samples. *n* = 6 per group. Unpaired *t*-test. *p < 0.05, **p < 0.01, ***p < 0.001, ****p < 0.0001, ns, not significant.

We next used Venn diagrams to determine which genes show consistent sexually dimorphic expression in neurons, astrocytes, and microglia and identified five such genes as *Ddx3y*, *Eif2s3y*, *Kdm5d*, *Uty*, and *Gm21975* (Figure 3A). The formal four identified genes locate on the Y chromosome and have relatively medium (FPKM > 1) to high (FPKM > 10) expressions in all three cell types of male origin (Figure 3B and Supplementary Figure 2). Protein-protein interaction (PPI) network analysis showed a robust interaction within the four Y-chromosomal genes (Figure 3C). The *Gm21975* gene (also known as *Evi2*) locates on chromosome 11. *Gm21975* expresses a naturally occurring read-through transcript covering the neighboring *Evi2a* (ecotropic viral integration site 2a) and *Evi2b* (ecotropic viral integration site 2b) genes and encodes a protein identical to the *Evi2b* gene product. However, although RNA sequencing results showed that *Gm21975* was expressed significantly higher in male samples than female samples, quantitative real-time PCR (qRT-PCR) validation found lower expression of *Gm21975* in male astrocytes than female astrocytes, and no *Gm21975* expression differences between male and female samples of neurons and microglia (Supplementary Figures 3A–C). Therefore, we excluded *Gm21975* from subsequent analysis.

We also validated the four identified Y-chromosomal genes by qRT-PCR. Consistent with RNA sequencing data, *Ddx3y*, *Eif2s3y*, *Kdm5d*, and *Uty* were solely expressed in neurons, astrocytes, and microglia of male origin, at levels dramatically higher in males than females (Figures 3D–F). Since all these genes have respective homologs on the X chromosome (*Ddx3x* for *Ddx3y*, *Eif2s3x* for *Eif2s3y*, *Kdm5c* for *Kdm5d*, and *Utx* for *Uty*), we speculated that their X chromosomal homologs might have a biased expression in females for functional compensation purpose. Indeed, qRT-PCR analysis revealed that *Kdm5c* and *Utx* were expressed significantly higher in all the three cell types of female origin than those of male origin (Figures 3D–F). However, *Ddx3x* was expressed only significantly higher in female astrocytes than male astrocytes; and *Eif2s3x* was expressed only significantly higher in astrocytes and microglia of female origin than those of male origin. Together, these results suggest that sexually dimorphic genes dominantly locate on sex chromosomes.

### Overexpression of *Eif2s3y* Increases sEPSC and sIPSC Amplitudes Specifically in Male Neurons

Given that *Eif2s3y* was relatively highly expressed compared to the other three Y-chromosomal genes in male neurons (Supplementary Figure 2) and that its X-linked homolog, *Eif2s3x* showed no significant differences in neurons between males and females (Figures 3B,D), we hypothesize that *Eif2s3y* plays a unique role in neuronal functions. Therefore, we studied whether overexpression of *Eif2s3y* affects neuronal synaptic transmission.

**FIGURE 4:**
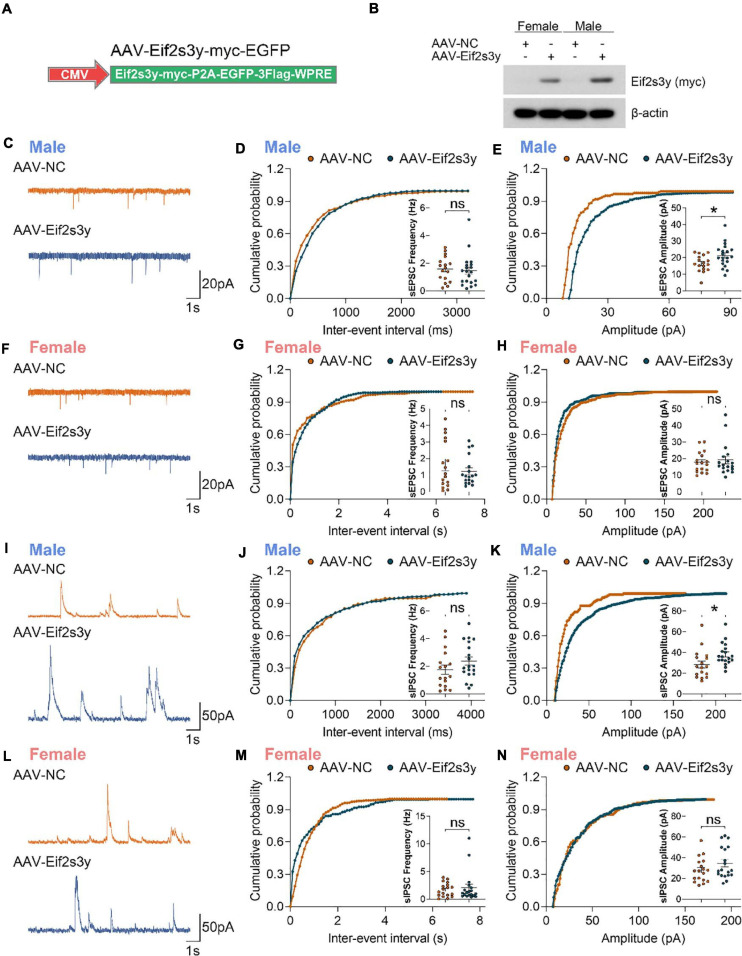
Overexpression of Eif2s3y promotes excitatory and inhibitory synaptic transmission specifically in male neurons. **(A)** Scheme of the Eif2s3y-Myc expressing construct used for AAV packaging. **(B)** Primary neurons from E16.5 male and female mice were infected with AAV expressing Eif2s3y (AAV-Eif2s3y) or EGFP (AAV-NC). Exogenous Eif2s3y protein was detected by western blotting using an anti-Myc antibody. **(C)** Representative traces of sEPSCs recorded from primary male neurons infected with AAV-Eif2s3y (*n* = 20 cells) and AAV-NC (*n* = 17 cells). Scale bars, 20 pA (vertical) and 1 s (horizontal). **(D,E)** Cumulative probabilities of sEPSC frequency **(D)** and amplitude **(E)** and respective quantification and comparison in male neurons. **(F)** Representative traces of sEPSCs recorded from primary female neurons infected with AAV-Eif2s3y (*n* = 18 cells) and AAV-NC (*n* = 17 cells). Scale bars, 20 pA (vertical) and 1 s (horizontal). **(G,H)** Cumulative probabilities of sEPSC frequency **(G)** and amplitude **(H)** and respective quantification and comparison in female neurons. **(I)** Representative traces of sIPSCs recorded from primary male neurons infected with AAV-Eif2s3y (*n* = 19 cells) and AAV-NC (*n* = 17 cells). Scale bars, 50 pA (vertical) and 1 s (horizontal). **(L,K)** Cumulative probabilities of sIPSC frequency **(L)** and amplitude **(K)** and respective quantification and comparison in male neurons. **(L)** Representative traces of sIPSCs recorded from primary female neurons infected with AAV-Eif2s3y (*n* = 19 cells) and AAV-NC (*n* = 18 cells). Scale bars, 50 pA (vertical) and 1 s (horizontal). **(M,N)** Cumulative probabilities of sIPSC frequency **(M)** and amplitude **(N)** and respective quantification and comparison in female neurons. Mann Whitney test was used for **(G,H,M)**. Unpaired *t*-test was used for **(D,E,J,K,N)**. *p < 0.05, ns, not significant.

We packaged AAVs that express the mouse *Eif2s3y* protein with a Myc-tag on the C-terminus (AAV-Eif2s3y) or an EGFP control (AAV-NC) (Figure 4A). Primary neurons from P0 male and female mice were infected with AAV-Eif2s3y or AAV-NC. *Eif2s3y* overexpression was confirmed by western blotting (Figure 4B) and qRT-PCR (Supplementary Figure 4). We performed whole-cell recordings in these primary neurons. We found that compared to controls, the amplitude but not the frequency of spontaneous excitatory postsynaptic currents (sEPSCs) (Figures 4C–E) and spontaneous inhibitory postsynaptic currents (sIPSCs) (Figures 4I–K) were significantly increased in male neurons overexpressing *Eif2s3y*. While in female neurons, neither the frequency nor the amplitude of sEPSCs (Figures 4F–H) and sIPSCs (Figures 4L–N) was affected by *Eif2s3y* overexpression.

### Overexpression of *Eif2s3y* Causes Autism-Like Behaviors Specifically in Male Mice

**FIGURE 5:**
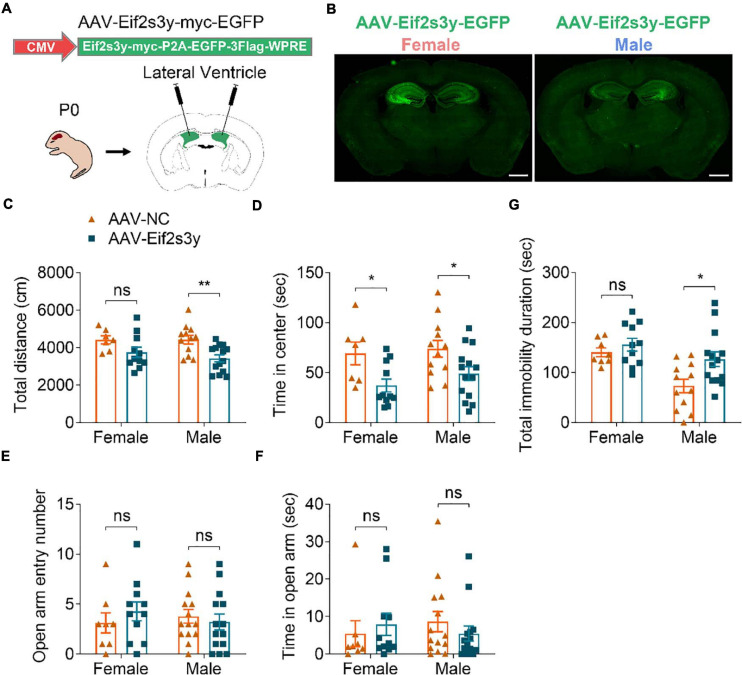
Overexpression of Eif2s3y causes male-specific depression-like behavior. **(A)** Scheme of the AAV injection paradigm. **(B)** Representative images of the EGFP expression localization in male and female mice injected with AAV-Eif2s3y. Scale bar, 1 mm. **(C,D)** In the open-field test, total travel distance **(C)** and time in the center **(D)** were analyzed. *n* = 14 AAV-Eif2s3y male mice, *n* = 12 AAV-NC male mice, *n* = 11 AAV-Eif2s3y female mice, and *n* = 7 AAV-NC female mice. **(E,F)** In the elevated plus-maze test, time spent in the open arm **(E)** and open arm entry numbers **(F)** were analyzed. *n* = 14 AAV-Eif2s3y male mice, *n* = 14 AAV-NC male mice, *n* = 11 AAV-Eif2s3y female mice, and *n* = 8 AAV-NC female mice. **(G)** In the tail suspension test, mouse immobility duration was analyzed. *n* = 14 AAV-Eif2s3y male mice, *n* = 12 AAV-NC male mice, *n* = 11 AAV-Eif2s3y female mice, and *n* = 8 AAV-NC female mice. Unpaired t-test. *p < 0.05, **p < 0.01, ns, not significant.

We next investigated whether Eif2s3y overexpression affects mouse behaviors. Both male and female neonatal C57BL/6 mice received a bilateral injection in the lateral ventricle of AAV-Eif2s3y or AAV-NC (Figure 5A). EGFP fluorescence observation revealed that AAVs infected largely the hippocampal and nearby regions (Figure 5B and Supplementary Figures 5A,B). Exogenous *Eif2s3y* protein expression was also detected in the hippocampus of both female and male mice injected with AAV-Eif2s3y compared to controls (Supplementary Figure 5C). In addition, we found that EGFP colocalized well with the neuronal marker NeuN but not with the astrocytic marker GFAP or the microglial marker Iba1 (Supplementary Figures 5D,E), indicating that AAVs mostly infected neurons, i.e., exogenous *Eif2s3y* was dominantly expressed in neurons.

Six weeks after AAV injection, we carried out various behavioral tests. In the open field test, *Eif2s3y* overexpression reduced total travel distance in male mice (Figure 5C) and decreased the time spent in the center region for both male and female mice (Figure 5D). However, in the elevated open arm test, *Eif2s3y* overexpression had no effect on the time spent in the open arm and open arm entries in either male or female mice (Figures 5E,F). These results suggest that *Eif2s3y* overexpression has a marginal effect on animal anxiety in both sexes. In the tail suspension test, *Eif2s3y* overexpression significantly increased immobility duration in male mice but not in female mice (Figure 5G), implying that *Eif2s3y* overexpression may specifically cause depression in male mice.

**FIGURE 6:**
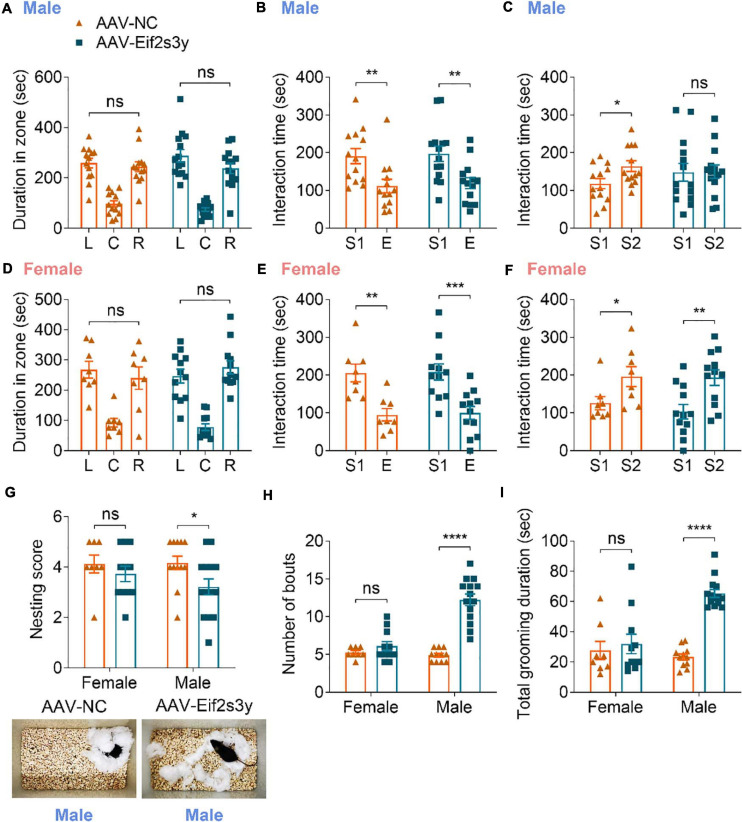
Overexpression of Eif2s3y causes autism-like behaviors specifically in male mice. **(A–F)** In the three-chamber social interaction test, male mice **(A–C)** injected with AAV-Eif2s3y (*n* = 14) or AAV-NC (*n* = 13), and female mice **(D–F)** injected with AAV-Eif2s3y (*n* = 12) or AAV-NC (*n* = 8) were tested in three steps. For habituation **(A,D)**, mice were compared for their time spent in each empty chamber (L, left; C, center; R, right). For sociability **(B,E)**, mice were compared for their interaction time with an empty wire cage (E) and with a mouse (S1). For social novelty recognition **(C,F)**, mice were compared for their interaction time with a familiar mouse (S1) and with an unfamiliar mouse (S2). **(G)** Both male and female mice were compared for their nest-building ability. Representative images of nests built by male mice are shown. *n* = 14 AAV-Eif2s3y male mice, *n* = 12 AAV-NC male mice, *n* = 11 AAV-Eif2s3y female mice, and *n* = 8 AAV-NC female mice. **(H,I)** During free movement, mice were analyzed for bout numbers **(H)** and total grooming duration **(I)**. *n* = 14 AAV-Eif2s3y male mice, *n* = 11 AAV-NC male mice, *n* = 11 AAV-Eif2s3y female mice, and *n* = 8 AAV-NC female mice. Unpaired t-test was used for **(A–F,I)**. Mann Whitney test was used for **(G,H)**. *p < 0.05, **p < 0.01, ***p < 0.001, ****p < 0.0001, ns, not significant.

In the three-chamber social interaction test, we found that *Eif2s3y* overexpression in male mice had no effect on their chamber exploration pattern during the habituation stage (Figure 6A) and their sociability to a mouse (S1) rather than to an empty cage (Figure 6B). However, *Eif2s3y* overexpression impaired the social novelty recognition of male mice to a familiar mouse (S1) versus an unfamiliar mouse (S2) (Figure 6C). In contrast, *Eif2s3y* overexpression in female mice had no effect on animal habituation, sociability, or social novelty recognition (Figures 6D–F). In the nest-building test, we also found that *Eif2s3y* overexpression specifically impaired nest building ability in male but not female mice (Figure 6G). Moreover, male but not female mice with *Eif2s3y* overexpression exhibited significantly increased bout numbers and grooming duration compared to controls (Figures 6H,I). Together, these results indicate that overexpression of *Eif2s3y* causes autism-like behaviors specifically in male mice.

**FIGURE 7:**
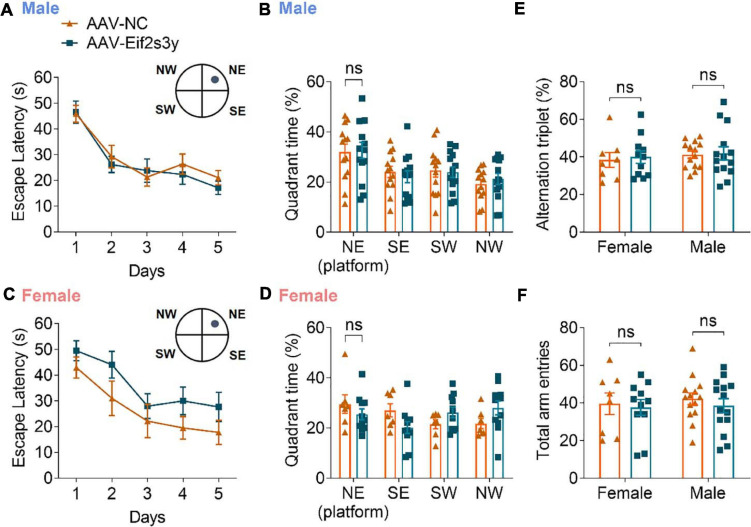
Overexpression of Eif2s3y has no effect on mouse learning and memory. **(A–D)** In the Morris water maze test, escape latency **(A,C)** in the training phase and time spent in each quadrant during the probe test phase **(B,D)** were analyzed for male mice **(A,B)** injected with AAV-Eif2s3y (*n* = 14) or AAV-NC (*n* = 13), and female mice **(C,D)** injected with AAV-Eif2s3y (*n* = 11) or AAV-NC (*n* = 7). Two-way ANOVA followed by *post hoc* Sidak’s multiple comparisons test was used for **(A,C)**. Unpaired t-test was used for **(B,D)**. ns, not significant. **(E,F)** In the Y maze test, alternation triplet **(E)** and total arm entries **(F)** were analyzed. *n* = 14 AAV-Eif2s3y male mice, *n* = 14 AAV-NC male mice, *n* = 11 AAV-Eif2s3y female mice, and *n* = 8 AAV-NC female mice. Unpaired *t*-test was used for **(E,F)**. ns, not significant.

We also studied whether *Eif2s3y* overexpression affects animal learning and memory. However, in the Morris water maze test, both male (Figures 7A,B) and female (Figures 7C,D) mice with *Eif2s3y* overexpression showed similar escape latencies during the training phase and similar time spent in the target quadrant during the probe test phase compared to respective controls. In the Y maze test, both male and female mice with *Eif2s3y* overexpression had similar alternation triplet and total arm entry numbers compared to respective controls (Figures 7E,F). Therefore, *Eif2s3y* overexpression may not affect learning and memory in mice.

Finally, we explored whether *Eif2s3y* overexpression affects synaptic proteins. However, the results showed that *Eif2s3y* overexpression in male mice did not affect total levels (Supplementary Figures 6A,B) and synaptic distribution (Supplementary Figures 6C-F) of proteins studied here, including AMPA receptor subunits (GluR1, GluR2, and GluR3), NMDA receptor subunits (NR1, NR2A, and NR2B), the presynaptic marker synaptophysin (SYP), and the postsynaptic marker PSD-95.

## Discussion

Sex differences have been documented in multiple neurological disorders, but underlying mechanisms remain largely elusive. Primary neural cells have been widely used as *in vitro* models for investigating cellular and molecular mechanisms of neurological disorders. However, the sex of donors of these primary cells is neglected in significant amounts of such studies; and this could potentially affect the correctness of their conclusions and be a source for contradictory results of different studies. Therefore, to determine whether a significant gene expression difference exists between male and female neural cells, we comprehensively compared the transcriptome of primary neurons, astrocytes and microglia derived from neonatal mice of the two sexes. We identified 69, 30, and 264 sexually dimorphic genes in primary neurons, astrocytes, and microglia, respectively. Compared to a total of 20,073 genes identified in this study, the numbers of sexually dimorphic genes in these cells are relatively low. Among the three cell types, microglia exhibit the most divergent expression differences between the two sexes, which is consistent with previous reports showing that microglia display sex differences both in their transcriptome and function (Hanamsagar et al., 2017; Guneykaya et al., 2018; Thion et al., 2018; Villa et al., 2018).

Gene ontology and KEGG analysis revealed that identified sexually dimorphic genes in neurons, astrocytes, and microglia exhibited similar annotation patterns. Interestingly, sexually dimorphic genes were strongly enriched for the immune system and immune-related diseases in all three cell types, suggesting that immune systems are the most divergent between males and females in the central nervous system (CNS). This finding is in agreement with many previous studies suggesting that immune systems are involved in the sex-specific etiology of multiple neurological disorders (Crain et al., 2009, 2013; Schwarz et al., 2012; Lenz et al., 2013; Dunn et al., 2015; Guneykaya et al., 2018; Villa et al., 2018).

Our RNA sequencing results identified five genes showing consistent sexually dimorphic expression in neurons, astrocytes, and microglia. Among them, four genes (*Ddx3y*, *Eif2s3y*, *Kdm5d*, and *Uty*) locate on the short arm of the mouse Y chromosome. This region is part of the Y-chromosomal non-recombining region so that genes on this region are present and expressed exclusively in males (Armoskus et al., 2014). Previous studies also identified sexually dimorphic expression of *Ddx3y*, *Eif2s3y*, *Kdm5d*, and *Uty* in the mouse brain at different ages by using northern blotting and RT-PCR (Xu et al., 2002), and of *Ddx3y*, *Eif2s3y*, and *Kdm5d* in the neonatal mouse cortex/hippocampus by using microarray analysis (Armoskus et al., 2014). *Ddx3y* encodes an RNA helicase containing a DEAD-box motif and has been implicated in RNA metabolism, neuronal regulation, apoptosis, and cell cycle progression (Vakilian et al., 2015). *Eif2s3y* is suggested to play a crucial role in mouse spermatogenesis (Mazeyrat et al., 2001; Yamauchi et al., 2014; Matsubara et al., 2015). *Kdm5d* encodes a histone demethylase and participates in H3K4 demethylation (Bernstein et al., 2002). *Uty* and its X-homolog *Utx* (also known as *Kdm6a*) belong to the KDM6 subfamily. *Utx* encodes a histone H3 lysine 27 (H3K27) demethylase. However, human *UTY* has been reported to lose enzymatic activity due to sequence divergence (Shpargel et al., 2012).

*Ddx3y*, *Eif2s3y*, *Kdm5d*, and *Uty* have their homologs on the X chromosome as *Ddx3x*, *Eif2s3x*, *Kdm6a*, and *Utx*, respectively. We found that *Kdm5c* and *Kdm6a* were expressed higher in all three female cell types than male cells, *Eif2s3x* was expressed higher in female astrocytes and microglia than male cells, and *Ddx3x* was expressed only significantly higher in female astrocytes than male astrocytes. These results imply that these X-chromosomal genes escape from X chromosome inactivation in certain female cell types, possibly as compensation for balancing the function of their Y-chromosomal homologous genes.

*Eif2s3y* was found to have a high expression in male neurons. However, the function of *Eif2s3y* in the CNS remains unknown. To study whether dysregulation of *Eif2s3y* affects neuronal functions in a sex-specific manner, we infected primary mouse neurons with AAVs expressing mouse *Eif2s3y* and found that overexpression of *Eif2s3y* increased the amplitude of both sEPSCs and sIPSCs in male but not female neurons. These results indicate that overexpression of *Eif2s3y* promotes both excitatory and inhibitory synaptic transmission specifically in males.

Moreover, we overexpressed *Eif2s3y* in the mouse brain and found that overexpression of *Eif2s3y* led to autism-like behaviors specifically in male mice, including impaired social novelty recognition and nest-building abilities and increased repetitive behavior. Since the protein sequence of mouse *Eif2s3y* is highly conserved (over 97% similarity) with those of mouse Eif2s3x and human X-linked EIF2S3, and there is no Y-linked homolog of *Eif2s3y* in humans (Supplementary Figures 7A,B), overexpression of *Eif2s3y* in male and female mice may partially resemble patients with Klinefelter Syndrome (47,XXY) and XXX Syndrome (47,XXX), respectively. Indeed, Klinefelter Syndrome patients have been reported to have an increased risk of ASDs (Visootsak and Graham, 2009; Tartaglia et al., 2010; Bishop et al., 2011; Hong and Reiss, 2014; Green et al., 2019); and our results suggest that an increased expression of EIF2S3 may at least partially contribute to this.

The human homolog of *Eif2s3y*, *EIF2S3* encodes eukaryotic translation initiation factor 2 subunit γ (eIF2γ) that plays a regulatory role in Met-tRNAi^Met^ binding and thus is involved in early protein synthesis (Young-Baird et al., 2019). Since dysregulation of protein synthesis has been found in ASD patients and animal models and proposed as a common mechanism underlying disease pathogenesis (Levitt and Campbell, 2009; Lo and Lai, 2020; Lu and Hsueh, 2021), it is possible that a dysregulation of *Eif2s3y* may promote susceptibility to ASD through interfering with the synthesis of proteins critically involved in ASD pathogenesis; and this warrants further investigation.

In summary, our present study provides a novel and unbiased sexually dimorphic gene expression dataset in primary neurons, astrocytes, and microglia derived from female and male neonatal mice. Our study also indicates that sexually dimorphic genes on the Y chromosome may be a genetic driver of neurological disorders, which deserves further scrutiny.

## Data Availability Statement

The datasets presented in this study can be found in online repositories. The names of the repository/repositories and accession number(s) can be found below: CNGB Sequence Archive (CNSA) of China National GeneBank DataBase (https://ftp.cngb.org/pub/CNSA/data3/CNP0001675/), accession number CNP0001675.

## Ethics Statement

The animal study was reviewed and approved by the Animal Ethics Committee of Xiamen University.

## Author Contributions

MZ and Y-WZ designed the research. MZ performed most experiments. YZ contributed to virus experiments. YJ, ZL, XX, and JN helped with animal experiments. XX helped with sample preparation for RNA sequencing. HS helped with electrophysiological experiments. XZ, HL, DC, JL, and HX made intellectual contributions. MZ, ZL, and Y-WZ wrote the manuscript. Y-WZ supervised the project. All authors reviewed the final manuscript.

## Conflict of Interest

The authors declare that the research was conducted in the absence of any commercial or financial relationships that could be construed as a potential conflict of interest.

## Footnotes

1 https://github.com/BGI-flexlab/SOAPnuke
