# Supplementary material for: Profiling of Sexually Dimorphic Genes in Neural Cells to Identify Eif2s3y, Whose Overexpression Causes Autism-Like Behaviors in Male Mice
Source: Front Cell Dev Biol. 2021 Jul 6;9:669798. doi: 10.3389/fcell.2021.669798 (PMC8292149; doi:10.3389/fcell.2021.669798)
Supplement: Supplementary file 1 [file Table_1.docx]

**Supplementary Table 1. Primers used for quantitative real-time PCR (qRT-PCR)**

| **Target gene** | **Forward Primer (5’-3’)** | **Reverse Primer (5’-3’)** |
| --- | --- | --- |
| ***Ddx3y*** | CCTTGGACTTGCCACCTCTT | AGAACGTCCACGGCTACTTC |
| ***Ddx3x*** | CAGCAAGCAAAGGGCGTTAT | CTTCTATCTCCACGGCCACC |
| ***Eif2s3y*** | TGACCCAACGTTATGCCGAG | AGGAGCCGTCTCAGTAGGAA |
| ***Eif2s3x*** | GGTGAGGGTGGAGTGACTCT | TTCCCATGAGCTACGTGACCA |
| ***Kdm5d*** | CCAGGATCTGACGACTTTCTACC | TTCTCCGCAATGGGTCTGATT |
| ***Kdm5c*** | ACCCACCTGGCAAAAACATTGG | ACTGTCGAAGGGGGATGCTGTG |
| ***Uty*** | CTGCTGCCCTAGGTAATGAGG | TCCACAAAGCGCCTTCTTCT |
| ***Kdm6a*** | CGGGCGGACAAAAGAAGAAC | CATAGACTTGCATCAGATCCTCC |
| ***Gm21975*** | TGGGCAGGTAGGTCTCCATT | TGAGACAACTGATGCACGCT |
| ***β-actin*** | AGCCATGTACGTAGCCATCCA | TCTCCGGAGTCCATCACAATG |
