# Supplementary material for: Profiling of Sexually Dimorphic Genes in Neural Cells to Identify Eif2s3y, Whose Overexpression Causes Autism-Like Behaviors in Male Mice
Source: Front Cell Dev Biol. 2021 Jul 6;9:669798. doi: 10.3389/fcell.2021.669798 (PMC8292149; doi:10.3389/fcell.2021.669798)
Supplement: Supplementary file 3 [file Table_3.docx]

**Supplementary Table 3. Differentially expressed genes between males and females identified in neurons, astrocytes, and microglia^*^**

| Neurons | Up | Rps27rt, Gm37013, Spon2, Gm13092, Slc6a20a, Gm6851, Gm45978, Hmga1b, Angpt2, Apod, Cdh5, Cfh, Col3a1, Dcn, Evi2a, Fcgr2b, Foxf2, Slc6a13, Gjb6, H3c14, Foxd1, Igf2, Itih2, Krt17, Lum, Tlr8, Mid1, Slc22a6, Aldh1a2, Slc22a8, Tbxas1, Evi2b, Tgm2, Trpc2, Ttr, Ugt1a2, Rpl10l, Ggt5, Ugt1a7c, Eno1b, Lrrc17. |
| --- | --- | --- |
|  | Down | Gm20815, Gm15319, Gm14308, Gm4567, Gm10651, Gm21996, Gm21064, Ccl21d, Evi2, LOC102640292, Gm37416, Gm41476, Gm21088, Gm50614, Gm21477, Gm21794, Gm21198, Eomes, Kdm5d, Uty, Wnt8b, Capn11, Ddx3y, Eif2s3y, Tfap2e, Gm20747, Hnrnpa1l2-ps2, Ccl21c. |
| Astrocytes | Up | Ppbp, Rps27rt, Gm3696, Gm6434, Gm40378, Gm13278, LOC101055676. |
|  | Down | Mup8, Mup19, Evi2, Alb, Kdm5d, Serpina1c, Uba1y, Uty, Aldob, Ddx3y, Eif2s3y, H3c11, Trim34b, Npcd, Col25a1, Gm9265, Gm2436, Gm21992, Gm33887, Gm40525, Gm45978, Gm46223, LOC100041057. |
| Microglia | Up | Kazald1, Bdkrb1, Cyp1a1, Dcc, Gdf10, Krt17, Nppc, Saa3, Sprr1a, Ugt8a, Igsf21, Rpl10l, Wif1, Adamts16, H3c2, H2ac6, H2bc22, H2ac19, Foxo6, Xlr4a, Tnfsf14, Gm13277, Gm6987, Armh4, Rsc1a1, Tcerg1l, Cda, Crtac1, Slc25a31, Fkbp6. |
|  | Down | Ifi208, Isg15, LOC100039029, Msmp, Tgtp2, Gm2666, Mndal, LOC100041057, Ly6c2, LOC100041903, Gm15433, LOC100503923, Gbp6, Gm21064, Cfap99, LOC100862473, LOC101055663, LOC101055672, Gm7592, Gm29804, Evi2, Gm4631, LOC102638047, LOC102638435, A530040E14Rik, Gm38510, Gm35498, lfi206, Gm38525, LOC102641031, Adcy4, Gm38699, Gm40498, Gm40525, Gm40991, Gm41476, LOC105247075, Gm42368, Gm6851, Gm45935, Nt5c3, Gm2619, LOC108168691, Epsti1, Apol7c, Sp110, Gpr18, Ly6a, H2-Q6, H2-Q9, Csprs, Aif1, Cd5l, Bmp10, Cd69, Cd72, Ccr2, Ccr7, Cyp2a4, Ddx4, Dhh, Dll1, Dnase1l3, Fcgr1, Fgl2, Il4i1, Flt4, Slc6a13, Gbp2b, Gbp2, Lrp2, Cfb, H2-Oa, H2-Q7, H2-T22, H2-T24, Hdc, Hrh2, Irgm1, Cxcl10, Ifi203, Ifi204, Ifi47, Ifit1, Ifit2, Ifit3, Ifnb1, Il18bp, Igtp, Il7, Acod1, Kdr, Klk1b11, Klra2, Lst1, Ly6c1, Ly6e, Bco2, Clec10a, Gbp4, Nos2, Ogn, Olfr56, Rhox5, Pla2r1, Pou3f1, Rasgrp1, Ccl12, Ccl4, Ccl5, Ccl7, Sell, Slc10a1, Slfn1, Slfn4, Kdm5d, Serpina3g, Spta1, Stat1, Stat2, Hsh2d, Sectm1a, Klhdc8a, Tap1, Calhm6, Cd300e, Tgtp1, Phf11a, Phf11d, Tnfrsf17, Cd40, Tnfsf8, Tpst1, Tnfsf10, Cmpk2, Uty, Apol9a, Enpp4, Gm4841, Ifi205, Gpr55, Gbp5, Gbp7, Themis2, Oasl1, Ppm1n, Ddx60, Ifi209, Phf11b, Gbp9, Slfn9, Aplnr, Serpina3f, Tlr11, Gm4951, Tnfsf18, Usp18, Trim30b, Fcgr4, Oas3, Il27, Fgd2, Capn11, Ddx3y, Eif2s3y, Slc28a2, Clec4a1, Klrk1, Timd4, Slco1a4, Ipcef1, Tnfsf15, Scimp, Xaf1, A530064D06Rik, Treml2, Arhgef37, Rbm44, Gpr141, Tmem171, A530032D15Rik, Ifi211, 8030474K03Rik, Gm5431, Lrrc14b, Sp140, Adgre4, Irf7, Irgm2, Ifi214, LOC546061, Gbp3, Amotl2, Ube2l6, Ly6i, Isg20, Rsad2, Zbp1, Ms4a4b, Iigp1, Ifi213, BC147527, Gbp10, Ptchd4, Phf11, Phf11c, Heatr9, LOC630751, BC023105, Gm12250, Ms4a4c, Gm7609, Ifit3b, Ifit1bl1, Herc6, LOC671917, Fibin, Bst2, Ms4a6b, Nectin4, Apol9b, 1600014C10Rik, Clec4a3, Vgll3, Ms4a6c, P2ry13, Batf2, Mlkl, Slamf7, Rnase6, Misp, Tlr9, Igsf9, Tinagl1, Slamf9, Ifi44. |

^*^Up: upregulated in females compared to males. Down: downregulated in females compared to males.
