# Supplementary material for: Profiling of Sexually Dimorphic Genes in Neural Cells to Identify Eif2s3y, Whose Overexpression Causes Autism-Like Behaviors in Male Mice
Source: Front Cell Dev Biol. 2021 Jul 6;9:669798. doi: 10.3389/fcell.2021.669798 (PMC8292149; doi:10.3389/fcell.2021.669798)
Supplement: Supplementary file 4 [file Image_1.pdf]

SUPPLEMENTARY FIGURES

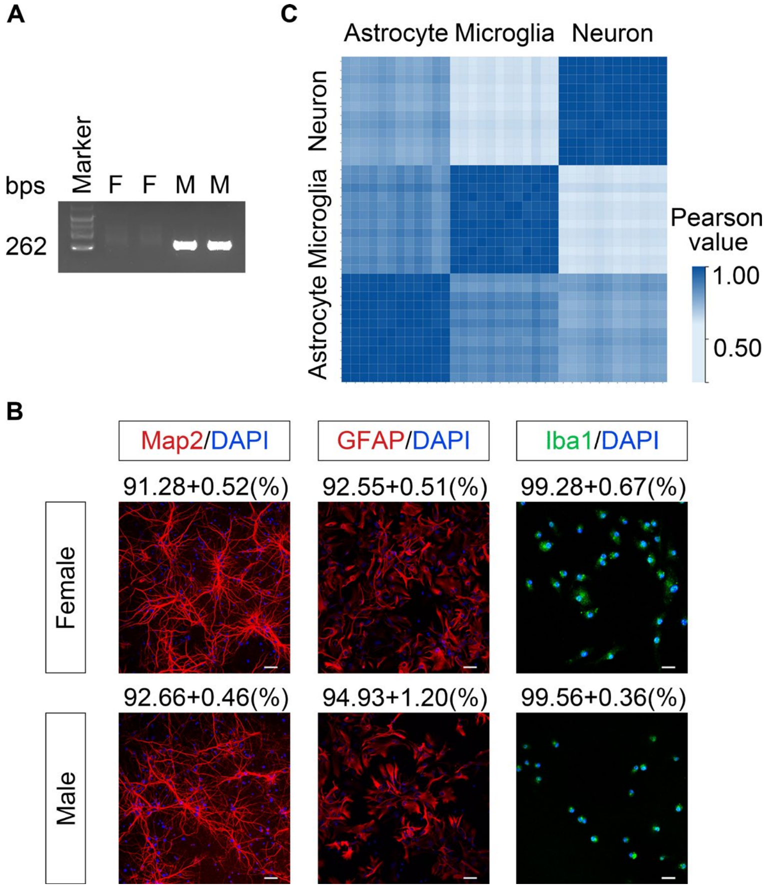

Supplementary Figure 1. Isolation of neurons, astrocytes, and microglia for RNA sequencing. **(A)** PCR genotyping of male (M) and female (F) mice. **(B)** Cultured primary neurons, astrocytes, and microglia from both P0 male and female mice were immunostained with Map2, GFAP, and Iba1, respectively, and stained with DAPI. Images were acquired by confocal microscopes to determine cell purity**.** The purity of each harvested cell culture was over 90%. Scale bars, 50 µm for neuron and astrocyte images, and 25 µm for microglia images. **(C)** Pearson's correlation analysis of samples subjected to RNA sequencing.

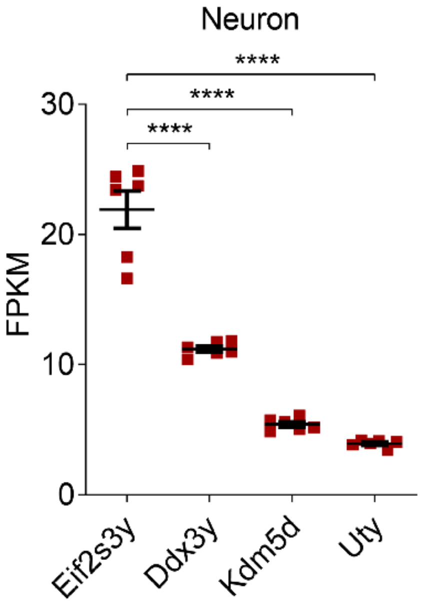

Supplementary Figure 2. *Eif2s3y* is highly expressed in male neurons. Expression of the four identified Y-chromosomal genes in neurons were compared based on their FPKM values. n = 6, one-way ANOVA followed by *post hoc* Dunnett's multiple comparisons test. ****$p < 0.0001$.

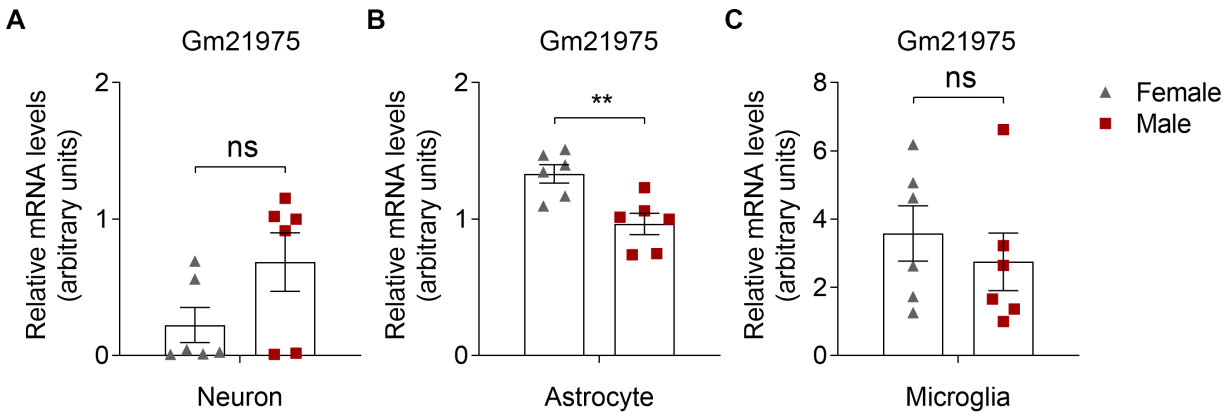

Supplementary Figure 3. Comparison of *Gm21975* expression between male and female samples. **(A-C)** *Gm21975* mRNA levels in neurons **(A)**, astrocytes **(B)**, and microglia **(C)** derived from P0 female and P0 male mice were measured by qRT-PCR for comparison. n = 6 per group, unpaired t-test. ** $p < 0.01$, ns, not significant.

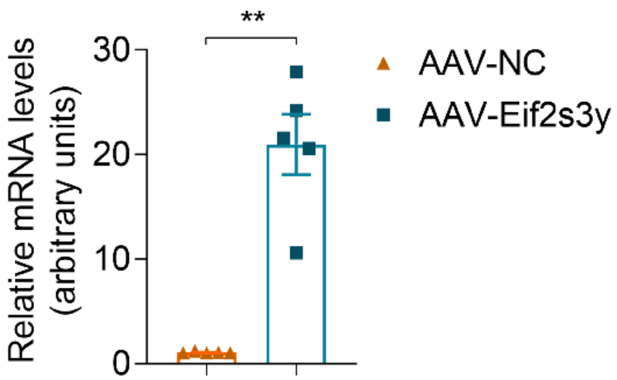

Supplementary Figure 4. Confirmation of *Eif2s3y* overexpression in AAV-infected neurons. Primary neurons from E16.5 male mice were infected with AAV-Eif2s3y or AAV-NC. *Eif2s3y* mRNA levels in neurons were measured by qRT-PCR for comparison. n = 5, Mann Whitney test. $^{**}p < 0.01$.

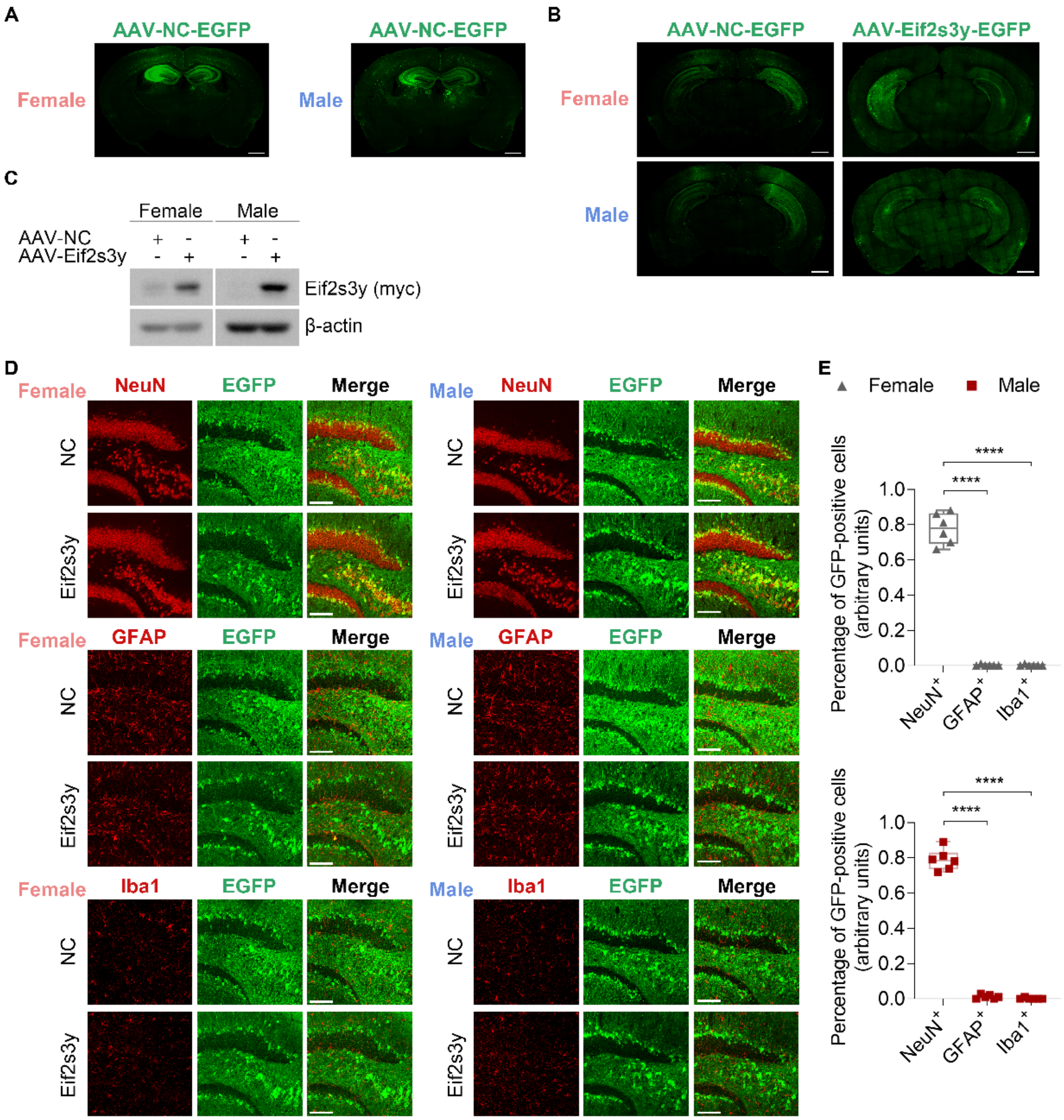

Supplementary Figure 5. Overexpression of Eif2s3y in mice. **(A)** Representative images in the dorsal hippocampal region of the EGFP expression localization in male and female mice injected with control AAVs (AAV-NC). Scale bar, 1 mm. **(B)** Representative images in the ventral hippocampal region of the EGFP expression localization in male and female mice injected with AAV-Eif2s3y or AAV-NC. Scale bar, 1 mm. **(C)** Exogenous Eif2s3y protein in the hippocampus

of both male and female mice injected with AAV-Eif2s3y was detected by western blotting. **(D)** Brain sections from both male and female mice injected with AAV-NC and AAV-Eif2s3y were subjected to immunostaining with the neuronal marker NeuN, the astrocytic marker GFAP, or the microglial marker Iba1. Hippocampal images were acquired by confocal microscopes to study the colocalization between EGFP and NeuN, GFAP, and Iba1. Scale bar, 100 μm. **(E)** Comparison of colocalization between EGFP and NeuN, GFAP, and Iba1 in **D**. n = 6 per group from three male and three female mice. One-way ANOVA followed by *post hoc* Dunnett's multiple comparisons test. ****$p < 0.0001$.

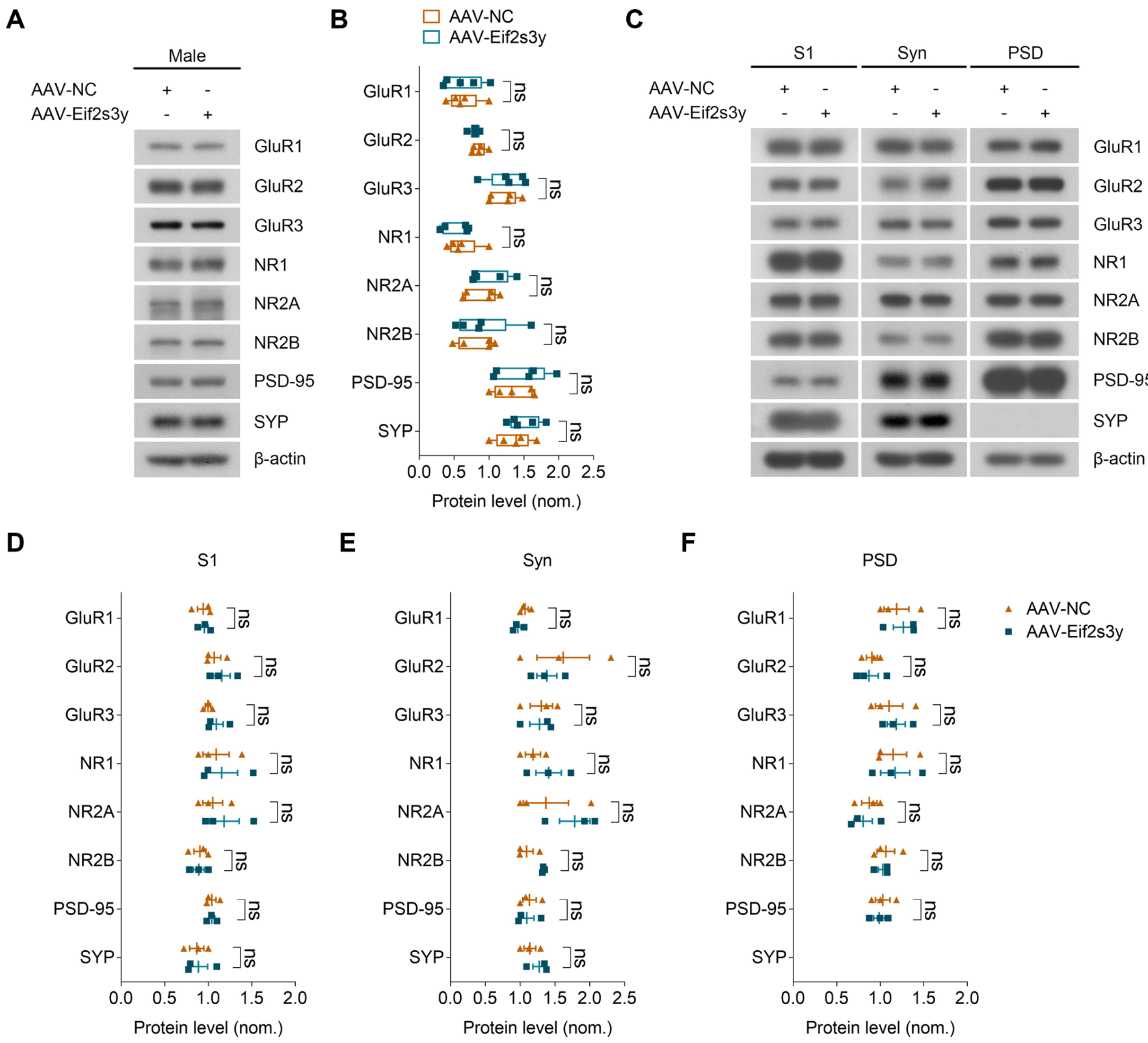

Supplementary Figure 6. Overexpression of Eif2s3y does not affect synaptic protein levels in male mice. **(A, B)** Indicated synaptic proteins in total lysates of the hippocampus of male mice injected with AAV-Eif2s3y and AAV-NC were detected by western blotting **(A)**. Protein band intensities were quantified for comparison **(B)**. n = 5 mice per group. Unpaired t-test. ns, not significant. **(C-F)** Indicated synaptic proteins in postnuclear (S1), synaptosomal (Syn), and post-synaptic density (PSD) fractions isolated from the hippocampus of male mice injected with AAV-Eif2s3y and AAV-NC were detected by western blotting **(C)**. Protein band intensities in S1 **(D)**,

Syn **(E)**, and PSD **(F)** fractions were quantified for comparison. n = 3 mice per group. Unpaired t-test. ns, not significant.

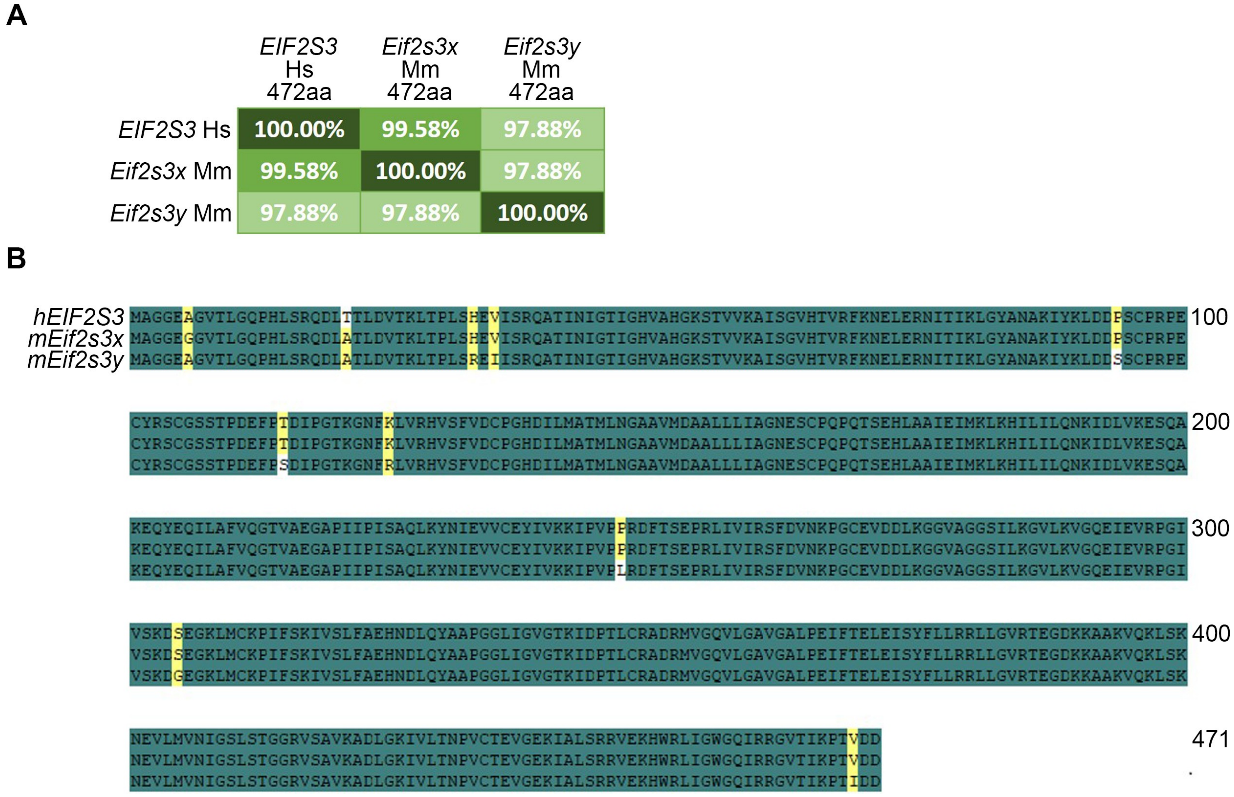

Supplementary Figure 7. Sequence comparison of human EIF2S3, mouse Eif2s3x, and mouse Eif2s3y proteins. **(A)** Amino acid identity comparison of EIF2S3, Eif2s3x, and Eif2s3y. Hs, human. Mm, mouse. **(B)** Protein sequence alignments of EIF2S3, Eif2s3x, and Eif2s3y. Identical amino acids were on a blue background.
